# Field and Current Controlled Domain Wall Propagation in Twisted Glass-Coated Magnetic Microwires

**DOI:** 10.1038/s41598-019-42352-1

**Published:** 2019-04-10

**Authors:** S. Corodeanu, H. Chiriac, A. Damian, N. Lupu, T.-A. Óvári

**Affiliations:** National Institute of Research and Development for Technical Physics, Iași, Romania

## Abstract

The torsion effect on the field and current driven magnetization reversal and the associated domain wall velocity in cylindrical amorphous and nanocrystalline glass-coated microwires is reported. Samples from three representative compositions have been investigated: (1) amorphous Fe_77.5_Si_7.5_B_15_ with positive magnetostriction, *λ* ≅ 25 × 10^−6^, (2) amorphous Co_68.18_Fe_4.32_Si_12.5_B_15_ with nearly zero negative magnetostriction, *λ* ≅ −1 × 10^−7^, and (3) nanocrystalline Fe_73.5_Si_13.5_B_9_Cu_1_Nb_3_ (FINEMET) with small positive magnetostriction, *λ* ≅ 2.1 × 10^−6^, all having the diameter of the metallic nucleus, *d*, of 20 µm and the glass coating thickness, *t*_*g*_, of 11 µm. The results are explained through a phenomenological interpretation of the effects of applied torque on the anisotropy axes within the microwires with different characteristics. Among all the complex mechanical deformations caused by the application of torque on magnetic microwire samples, the most important are the axial compression – for axial field-driven domain wall motion, and the circumferential tension – for electrical current/circumferential field-driven domain wall motion. The Co_68.18_Fe_4.32_Si_12.5_B_15_ microwire, annealed at 300 °C for 1 hour and twisted at 168 Rad/m exhibits the optimum characteristics, e.g. the lowest switching current (down to 9 mA~2.9 × 10^−3^ A/cm^2^) and the largest domain wall velocity (up to 2300 m/s).

## Introduction

Nowadays, the domain wall (DW) dynamics in thin magnetic wires represents an extremely exciting topic for both fundamental research and applications, mainly as concerns the development of novel, miniaturized magnetic sensors and DW-based logic devices^1,2^. In the case of logic devices, a particular attention is given to the easy manipulation and propagation of the magnetic DWs within the magnetic materials which function as DW conduits^3,4^. In magnetic nanowires, the DW motion can be driven either by an applied external magnetic field or by a spin-polarized electric current^5–8^. The field-driven DW dynamics was extensively studied in many types of materials, including the amorphous glass-coated microwires which exhibit a large Barkhausen effect (LBE). In this type of magnetically bistable wires, the magnetization switches in a single step when the external field is reversed. In a completely saturated sample, the switching mechanism entails the nucleation of a new domain with reverse magnetization, which leads to the formation of a new 180° domain wall near one of the wire ends, or near a defect, followed by the spontaneous subsequent displacement of this DW along the wire axis^9–14^. In most cases, the 180° domain wall pre-exists near the wire end due to demagnetization, fact that considerably simplifies the magnetization switching mechanism, since a much smaller applied field is required to just push this DW along the wire. The velocity of the propagating DWs strongly depends on the magnitude of the driving magnetic field and on the wire characteristics, including composition, dimensions, microstructure, as well as the mechanical stresses induced during preparation (intrinsic), which originate in both the rapid quenching process and in the presence of the glass coating^15–18^. Externally applied mechanical stresses (extrinsic) also affect the domain wall velocity (DWV)^19,20^. Although extensively studied, the magnetic field driven DW motion in glass-coated microwires has relatively low applicability in developing logic devices, due to the difficulty of generating multiple magnetic fields on each magnetic wire segment. The current excitation, on the other hand, by passing current directly through the magnetic sample, appears to be simpler and more suitable for practical purposes. There are many reports on the current-driven domain wall motion in nanometric magnetic strips obtained by means of deposition^21,22^, but only a very few on the effects of a current passing through the sample on the magnetic properties of Co-based microwires^23,24^.

Here, we report on the torsion effect on the field and current driven hysteresis loop and the associated DWV in cylindrical amorphous and nanocrystalline glass-coated microwires with large positive magnetostriction (FeSiB), small positive magnetostriction (FeSiBCuNb) and nearly zero negative magnetostriction (CoFeSiB). The results have been explained through a phenomenological interpretation of the effects of applied torque on the anisotropy axes within the microwires with different characteristics.

## Results

The experiments have been performed on glass-coated microwires with typical dimensions: the diameter of the metallic nucleus, *d*, of 20 µm, and the glass coating thickness, *t*_*g*_, of 11 µm. Samples from three representative compositions have been investigated: (1) amorphous Fe_77.5_Si_7.5_B_15_ with positive magnetostriction, *λ* ≅ 25 × 10^−6^, (2) amorphous Co_68.18_Fe_4.32_Si_12.5_B_15_ with nearly zero negative magnetostriction, *λ* ≅ −1 × 10^−7^, and (3) nanocrystalline Fe_73.5_Si_13.5_B_9_Cu_1_Nb_3_ (FINEMET) with small positive magnetostriction, *λ* ≅ 2.1 × 10^−6^. All the analysed samples have been prepared by means of glass-coated melt spinning^25^ at the National Institute of Research and Development for Technical Physics in Iași, Romania. Fe_77.5_Si_7.5_B_15_ amorphous samples are magnetically bistable in the as-cast state, whereas Co_68.18_Fe_4.32_Si_12.5_B_15_ ones require annealing at moderate temperatures to induce bistability. On the other hand, FINEMET samples require a typical annealing to induce the nanocrystalline structure.

The Co_68.18_Fe_4.32_Si_12.5_B_15_ glass-coated microwire has been annealed at 300 °C for 1 hour in order to partially relieve the frozen-in mechanical stresses and to achieve the magnetically bistable behaviour^26^. The initially amorphous Fe_73.5_Si_13.5_B_9_Cu_1_Nb_3_ glass-coated microwire has been annealed at 550 °C for 1 hour in order to achieve the nanocrystalline state and to reduce the magnetostriction constant^27–30^.

**Figure 1:**
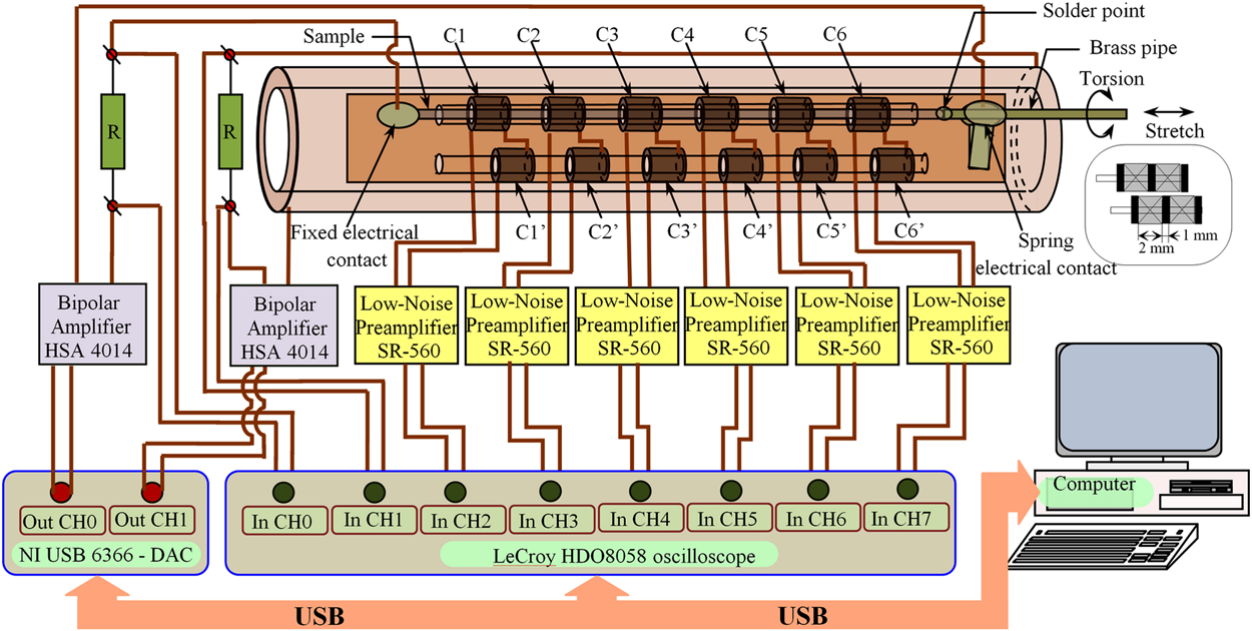
Schematic of the experimental system employed for the recording of the domain wall velocity under different conditions.

Due to the difficulties in monitoring the nucleation of multiple new domains with reversed magnetization and of the associated propagation of the newly formed 180° domain walls within the classical Sixtus-Tonks setup, we have developed a more complex system based on an enhanced variant of the Sixtus-Tonks method, that allows one to correctly identify the domain walls and to measure their DWV^31,32^, as well as to determine the axial hysteresis loop by an inductive method, in the case of both magnetic field or electric current driven magnetization reversal. A schematic of this system is illustrated in Fig. 1. Its key enhancement refers to the possibility of simultaneously passing a current and applying a torsion to a microwire sample.

The entire existence of the domain walls and of their movement within the wires demands the presence of an axially magnetized magnetic domain (easy axis parallel to the wire axis). The reversal of the magnetization within this domain looks like a jump and is always connected with the appearance of a rectangular hysteresis loop^33^.

**Figure 2:**
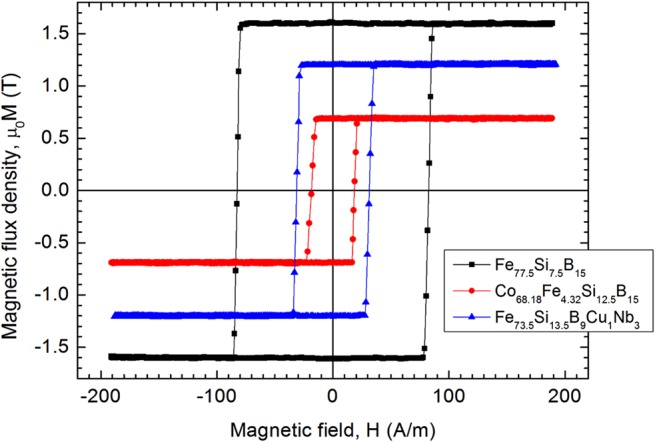
Hysteresis loop of the Fe_77.5_Si_7.5_B_15_ – as cast, Co_68.18_Fe_4.32_Si_12.5_B_15_ – annealed at 300 °C-1h and Fe_73.5_Si_13.5_B_9_Cu_1_Nb_3_ – annealed at 550 °C-1h glass-coated microwires with diameter of the metallic nucleus, d = 20 µm, and glass thickness, t_g_ = 11 µm.

In the as-cast amorphous state, both Fe_77.5_Si_7.5_B_15_ and Fe_73.5_Si_13.5_B_9_Cu_1_Nb_3_ glass-coated microwires display rectangular field-driven hysteresis loops, behaviour that is specific to the glass-coated amorphous microwires with positive magnetostriction^34^. The switching field values of both types of as-cast samples are about 80 A/m. The field-driven axial hysteresis loop of the Fe_73.5_Si_13.5_B_9_Cu_1_Nb_3_ glass-coated microwire remains rectangular even after nanocrystallisation, except the switching field of the sample decreases to about 30 A/m. The as-cast Co_68.18_Fe_4.32_Si_12.5_B_15_ amorphous glass-coated microwires do not exhibit rectangular field-driven axial hysteresis loop, the magnetization presenting a linear dependence on the axially applied magnetic field. This behaviour is specific to glass-coated amorphous microwires with negative magnetostriction and is due to the coupling between magnetostriction and internal stresses induced during preparation^25,35^. Annealing at 300 °C for 1 hour partially relieves the stresses and, as a consequence, the hysteresis loop becomes rectangular^26,36^. The measured switching field after annealing was 18 A/m. The axial field-driven hysteresis loops of the investigated samples are shown in Fig. 2.

Following our goal to control the magnetic domain wall movement by using an electric current that passes through the wires, we performed a series of experiments trying to identify the factors which influence the possibility to induce current-driven magnetization switching. First, we applied an AC current through the samples and we observed some low amplitude peaks in the induced signal which have proven to be due to the reorientation of the magnetic moments from the axial to the circumferential direction, determined by the circumferential magnetic field produced by the electric current. The maximum amplitude of the applied current was 240 mA (~764 A/mm^2^ for 20 µm diameter of the metallic core). At higher applied currents, the wire sample starts to deteriorate due to excessive heating.

**Figure 3:**
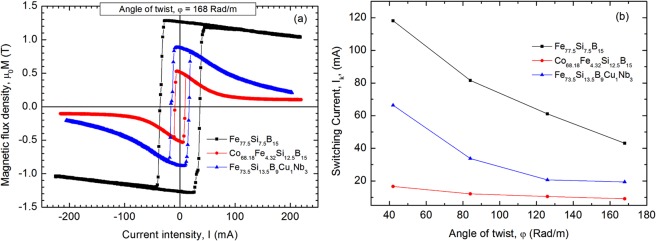
The hysteresis loop, defined as longitudinal magnetization change versus current intensity **(a)** and switching current versus wire torsion **(b)**, measured on the Fe_77.5_Si_7.5_B_15_ – as cast, Co_68.18_Fe_4.32_Si_12.5_B_15_ – annealed at 300 °C-1h and Fe_73.5_Si_13.5_B_9_Cu_1_Nb_3_ – annealed at 550 °C-1h glass-coated microwires with diameter of the metallic nucleus, d = 20 µm, and glass thickness, t_g_ = 11 µm.

When the samples are twisted, the induced peaks become sharper and their amplitudes increase significantly. We integrated the induced signals in order to obtain the sample’s change in axial magnetization, and we represented them versus the applied current. The resulting hysteresis loops (Fig. 3(a)) clearly show the axial magnetization switching by LBE, similar to the case of axial magnetic field driven switching (Fig. 2). The decrease in the axial magnetization when the current increases is due to the reorientation of the magnetic moments from the axial towards the circumferential direction under the influence of the circumferential magnetic field created by the current. The magnetization decreases more rapidly for the Co_68.18_Fe_4.32_Si_12.5_B_15_ sample, which means that the magnetization rotates more easily from the axial to the circumferential direction, possibly because of its larger circumferential permeability^37^.

Similar to the definition of the switching field for magnetic field excitation^16^, we defined in this case, for current excitation, the switching current (I_k_), as being the current above which the inner magnetic domain switches its magnetization from one direction to the opposite one by means of LBE. Figure 3(b) shows the evolution of the switching current versus applied torsion. The current at which LBE occurs decreases with the increase in the angle of twist for all the investigated samples. The smallest switching current has been observed in the case of the annealed Co_68.18_Fe_4.32_Si_12.5_B_15_ samples, specifically 17 mA at an angle of twist of 42 rad/m and dropping to 9 mA for an angle of twist of 168 rad/m.

**Figure 4:**
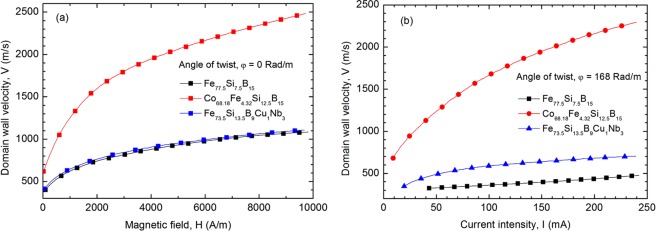
Domain wall velocity versus applied field **(a)** and current **(b)** measured on the Fe_77.5_Si_7.5_B_15_ – as cast, Co_68.18_Fe_4.32_Si_12.5_B_15_ – annealed at 300 °C-1h and Fe_73.5_Si_13.5_B_9_Cu_1_Nb_3_ – annealed at 550 °C-1h glass-coated microwires with diameter of the metallic nucleus, d = 20 µm, and glass thickness, t_g_ = 11 µm.

The field-driven DW displacement, in samples without torsion, shows an increase of the DWV with the increase of the AC excitation field amplitude for all the analysed samples (Fig. 4(a)). The current induced DW displacement has been only observed in twisted samples, and the DWV increases with the increase in the AC excitation current amplitude (Fig. 4(b)). The highest DWV values have been measured in both cases (axial field and current excitation) for the Co_68.18_Fe_4.32_Si_12.5_B_15_ samples annealed at 300 °C for 1 hour.

**Figure 5:**
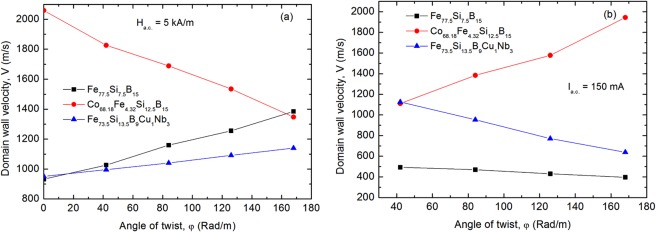
Domain wall velocity versus applied torsion for field (**a**) and current (**b**) excitation measured on the Fe_77.5_Si_7.5_B_15_ – as cast, Co_68.18_Fe_4.32_Si_12.5_B_15_ – annealed at 300 °C-1h and Fe_73.5_Si_13.5_B_9_Cu_1_Nb_3_ – annealed at 550 °C-1h glass-coated microwires with diameter of the metallic nucleus, d = 20 µm, and glass thickness, t_g_ = 11 µm.

Figure 5 shows the dependence of the DWV versus applied torsion for an AC applied field with the amplitude of 5 kA/m and for an AC applied current with the amplitude of 150 mA. The evolution of the DWV versus applied torsion is almost linear and changes its slope in opposite direction for field and current excitation and also for samples with different signs of the magnetostriction constant, respectively.

## Discussion

In order to analyse and understand the experimental results, we propose a phenomenological interpretation of the effects generated by the two driving forces that produce the magnetization reversal process: (i) the axially applied magnetic field which leads to the axial field-driven DW propagation, and (ii) the circumferential field created by the applied current, which determines the current-driven DW motion. For each individual trigger of magnetization reversal, one can address the case of positive and negative magnetostrictive samples, respectively.

**Figure 6:**
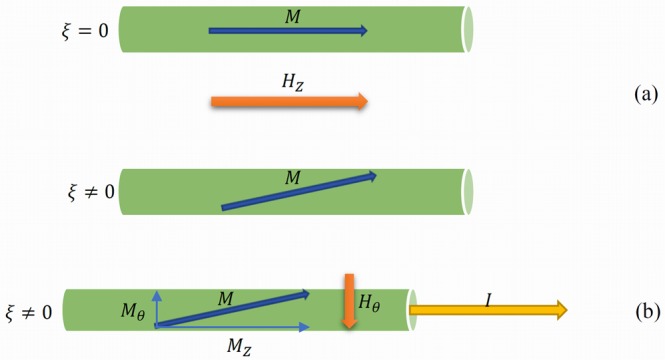
(**a**) Schematic of the wire magnetization subjected to axial field in the absence (top) and presence (bottom) of applied torque. (**b**) Current-driven magnetization reversal in amorphous microwire subjected to applied torque.

Hence, when the driving force of magnetization switching is the axial field, in the case of positive magnetostrictive samples (*λ* ≫ 0 and *λ* > 0, i.e. Fe_77.5_Si_7.5_B_15_ and FINEMET, respectively), one can identify two distinct situations, as shown in Fig. 6(a) below: (1) when the applied torque is null (*ξ* = 0), in which case the axial component of the magnetization is maximum, i.e. *M*_*z*_ = |*M*|, and (2) when there is a certain applied torque (*ξ* ≠ 0), in which case the axial component of the magnetization decreases as a result of the rotation of the easy axis out of the wire axis direction, towards the circular direction, i.e. *M*_*z*_ < |*M*|, which will result in a decrease of the axial magnetoelastic anisotropy *K*_*z*_.

In the second case (*ξ* ≠ 0), the axial anisotropy is given by:

1 $${K}_{Z}=\frac{3}{2}\lambda ({\sigma }_{Z}^{{\rm{intrinsic}}}+{\sigma }_{Z}^{{\rm{applied}}})$$

in which ${\sigma }_{Z}^{{\rm{intrinsic}}}$ is the axial component of the intrinsic mechanical stresses induced during preparation, being large and positive in the magnetically bistable core, whilst ${\sigma }_{Z}^{{\rm{applied}}}$ is the axial component of the extrinsic stress created by the applied torque. Therefore, the only way to decrease *K*_*z*_ in the core is if ${\sigma }_{Z}^{{\rm{applied}}}$ is negative (compressive). Thus, the most effective outcome of applied torque at the level of the magnetically bistable core in terms of mechanical action is an axial compression (${\sigma }_{Z}^{{\rm{applied}}} < 0$).

**Figure 7:**
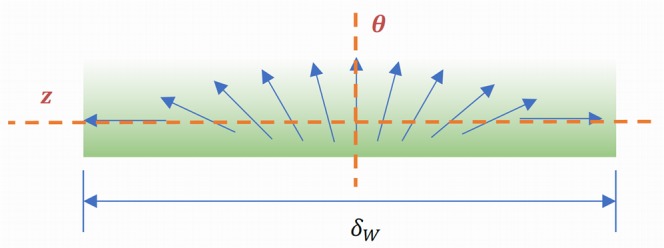
Representation of the 180° DW with the two orthogonal axes affected by the applied torque under various circumstances.

Now, this decrease of *K*_*z*_ with the applied torque *ξ* will allow faster propagation of the 180° DW (increased DWV) following magnetization switching under the action of the axial field. To grasp this statement, one has to go back to the actual meaning of the 180° DW, schematically represented below (Fig. 7), where *δ*_*W*_ stands for the width of the wall.

This 180° distribution of the spins inside the wall is analogue to a local perturbation that actually propagates along the wire axis, which means that, in the end, all the spins along the axis will rotate with 180°. Clearly, if the rotation is obstructed by a large axial anisotropy, such as the magnetoelastic one, *K*_*z*_, then, the local perturbation will propagate more slowly, resulting in smaller value of the DWV.

Therefore, a decrease of *K*_*z*_ similar to the one with applied torque in our case, will ultimately enhance the domain wall velocity in the samples with positive magnetostriction, such as the Fe_77.5_Si_7.5_B_15_ and FINEMET ones, as shown in Fig. 5(a).

When the driving force is the same (axially applied field), but the samples have negative magnetostriction (*λ* < 0), even though small, such as in the case of Co_68.18_Fe_4.32_Si_12.5_B_15_ microwires, the axial compression ${\sigma }_{Z}^{{\rm{applied}}} < 0$ will remain the most effective mechanical outcome of the applied torque *ξ* ≠ 0. However, in samples with low negative magnetostriction, the easy axis becomes parallel to the wire axis only after the annealing-induced stress relief. Therefore, the direction of the easy axis has a magnetostatic origin (shape anisotropy) and not a magnetoelastic one. The magnetoelastic term in these samples is weak from the beginning (as-cast state) due to the very small $\lambda$, and becomes even weaker after the stress relief annealing.

In this case, the axial compression produced by the applied torque, coupled with the negative magnetostriction, will add an extrinsic magnetoelastic component that will enhance the overall axial magnetic anisotropy in the bistable core:

2 $${K}_{Z}^{{\rm{total}}}={K}_{z}^{{\rm{shape}}}+{K}_{Z}^{{\rm{extrinsic}}}$$

with ${K}_{z}^{{\rm{extrinsic}}}=\frac{3}{2}\lambda {\sigma }_{Z}^{{\rm{applied}}}$, in which both *λ* and ${\sigma }_{Z}^{{\rm{applied}}}$ are negative.

Therefore, the total axial anisotropy increases with applied torque. This will obstruct the rotation of spins within the domain wall, and thus the propagation of the local perturbation (180° distribution of spins) will be more difficult, resulting in reduced velocities of the domain wall as the applied torque increases, as illustrated in Fig. 5(a).

Current-driven domain wall propagation occurs when the driving force of magnetization switching is the circumferential field *H*_*θ*_ produced by the current through a sample. In this case, the magnetization reversal process is triggered by the action of *H*_*θ*_ on the circumferential component of the magnetization *M*_*θ*_. This situation is schematically shown in Fig. 6(b). The circumferential component of the magnetization *M*_*θ*_ appears due to the applied torque *ξ* and increases as *ξ* increases.

The decrease of *K*_*z*_ due to applied torque in samples with positive magnetostriction, such as Fe_77.5_Si_7.5_B_15_ and FINEMET with *λ* ≫ 0 and *λ* > 0, respectively, does not enhance the domain wall propagation like in the case of field-driven magnetization reversal. This time, the reversal process takes place on the circumferential direction (*H*_*θ*_ → *M*_*θ*_) and, therefore, one should monitor the changes induced by the applied torque in the magnetic anisotropy on this particular direction, i.e. *K*_*θ*_. Nevertheless, the axial component of the magnetization *M*_*z*_, which we observe in our measurements, is dragged to switch by the reversing circumferential component *M*_*θ*_.

As the applied torque *ξ* increases, the circumferential component *M*_*θ*_ also increases, whereas the axial one *M*_*z*_ decreases (similar to the case in which positive magnetostrictive samples are switching under the action of the axial field).

Thus, *K*_*θ*_ increases with applied torque, which, due to the positive magnetostriction, can only occur if there is a tensile stress component on the circumferential direction (${\sigma }_{\theta }^{{\rm{applied}}} > 0$) caused by the torque. This circumferential tension increases with applied torque, as does the corresponding magnetoelastic anisotropy *K*_*θ*_.

The increased *K*_*θ*_ will make it more difficult for the spins to pass through the *θ* direction in their rotation, as illustrated in Fig. 7 above. This will hinder the propagation of the local perturbation in the orientation of the spins (the actual domain wall). Consequently, the DWV decreases with the increase in applied torque when the driving force is on the circumferential direction, as illustrated in Fig. 5(b) for the Fe_77.5_Si_7.5_B_15_ and FINEMET samples.

When the samples have small negative magnetostriction (Co_68.18_Fe_4.32_Si_12.5_B_15_) and the driving force is the circumferential field, the circumferential tension ${\sigma }_{\theta }^{{\rm{applied}}} > 0$, which is the most important mechanical consequence of applied torque on the circumferential direction, will produce an extrinsic magnetoelastic component on an orthogonal direction (due to *λ* < 0), but the anisotropy on the circumferential direction *K*_*θ*_ will decrease, and the larger the applied torque, the larger the reduction in *K*_*θ*_. This situation is somewhat similar to the more intuitive case of axially applied tensile stress in the case of samples with *λ* < 0: the larger the axial external tensile stress, the more the axial hysteresis loop is inclined, showing a strong reduction in the axial component of magnetization and in the axial anisotropy, accordingly. Here, there is a similar case, just on a different direction.

Thus, with *K*_*θ*_ decreasing as the applied torque *ξ* increases, the domain wall, whose propagation is driven by the action of the circumferential field on the circumferential component of the magnetization (*H*_*θ*_ → *M*_*θ*_), will be able to move more and more freely, increasing its velocity with torque, as shown in Fig. 5(b).

## Conclusions

The torsion effect on field and current driven LBE and DWV was investigated for the case of glass-coated microwires with three compositions having positive or negative magnetostriction constants. Axial magnetization switching by LBE similar to the one obtained by means of axial magnetic field excitation was clearly highlighted by axial magnetization vs. applied electrical current measurements. The current induced switching by LBE, along with the torsion dependence of the field and current-driven DWV, have been explained by taking into account the main extrinsic stresses induced by torsion, and their effect on the magnetic anisotropy of the microwires with different characteristics. Among all the complex mechanical deformations caused by the application of torque on magnetic microwire samples, the most important are the axial compression – for axial field-driven domain wall motion, and the circumferential tension – for electrical current/circumferential field-driven domain wall motion.

By comparing all the obtained results on the analysed samples, one can conclude that the Co_68.18_Fe_4.32_Si_12.5_B_15_ microwire, annealed for 1 hour at 300 °C and twisted at 168 Rad/m exhibits the optimum characteristics, e.g. the lowest switching current (down to 9 mA~2.9 × 10^−3^ A/cm^2^) and the largest DWV (up to 2300 m/s), which are useful for implementation in current-driven DW displacement related applications.

The current-driven magnetization switching of microwires could also be considered, with further refinement in terms of sample dimensions, for applications as novel magnetic logic/storage systems, as well as for the development of new sensing devices.

## Methods

The experimental system used to perform magnetic tests on the selected samples consists of a solenoid for magnetic field generation and six pairs of pickup coils secured on a rigid support (Fig. 1) which allows one to make electrical contacts at the sample ends and to apply torsional or tensile stress if required. The magnetizing solenoid (37 cm long, 2 cm in diameter, 2335 turns with a field to current constant of 6214 Am^−1^/A) is powered by a NI USB 6366 – digital to analog convertor (DAC) through a high-power bipolar amplifier HSA 4014.

The current through the sample is also applied by the same NI USB 6366 – DAC, on a different channel, through a second high-power bipolar amplifier HSA 4014. The digital to analog convertor allows two channels synchronous arbitrary wave form generation and was preferred to a regular function generator due to the virtually unlimited possibilities in choosing applied current and magnetic field variation modes and cross correlation between them. The second bipolar amplifier is used in order to obtain a voltage level high enough to inject a reasonable current through the high resistivity magnetic microwires^38^. The pickup coils are wound on two ceramic tubes with a 1 mm inner diameter, 1.8 mm outer diameter and 35 mm length. Each coil has 2 mm in width and 500 turns wound with enamelled 0.07 mm copper wire. The adjacent coils are separated by a 1 mm spacer, resulting in a distance between the centres of each adjacent coils of 3 mm. Each coil from one tube is connected in series-opposition with the corresponding one from the second tube. In this way, if the system is placed in a uniform alternating magnetic field, the signal induced in one coil is cancelled by the signal induced in its pair, resulting in a zero signal at the output of each pair. If a magnetic sample is inserted in one tube, then the signal at the output of each pair, in the presence of a uniform alternating field, will be given only by the fluctuation of the sample magnetization, the excitation field component being rejected due to coil compensation. In order to pass a current through the magnetic wire and to allow an easy change of the torsion angle, one end of the wire is soldered to a small brass pipe connected in the electrical circuit through a soft spring contact, to allow rotation or stretching, whilst the second wire end is firmly soldered to the rigid support of the pickup coil system. Due to the system configuration, the pickup coil detects only the sample magnetization rotations which have components along the wire axis (along the pickup coil axes direction), being essentially ‘blind’ to magnetization rotations in perpendicular planes.

**Figure 8:**
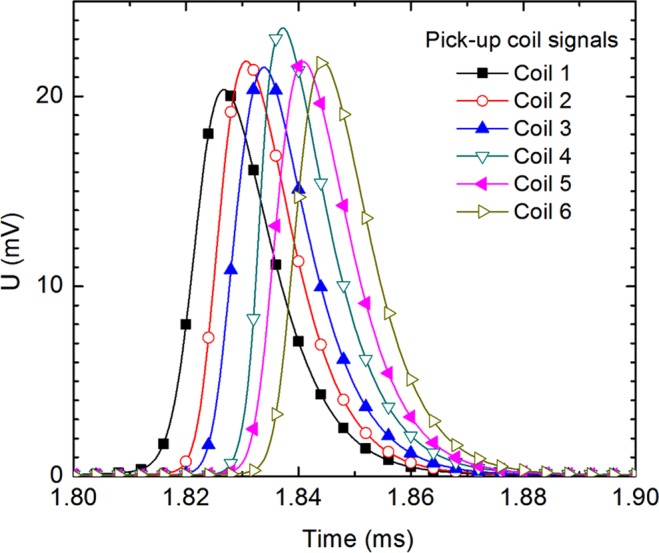
Consecutive peaks on the recorded signals used to compute domain wall velocity DWV.

For a soft magnetic wire showing LBE, the domain wall propagation along the wire is indicated by the appearance of consecutive peaks on the recorded signals, as shown in Fig. 8, with their order given by the coil number - C1, C2, …, C6 in the case of left to right propagation, and C6, C5, …, C1 for right to left propagation, according to Fig. 1.

The DWV is calculated as the ratio of the distance between two pick-up coils and the time difference between the induced signal peaks, corresponding to the considered coils.

The measurement accuracy of the DWV measuring system depends mainly on distance between the pick-up coils and on the distance measurement error. The overall error is estimated at 2% when measuring the propagation of a single DW on the entire length covered by the 6 pick-up coils.

The presented hardware design is completed by a specially designed LabView software, which allows the generation of signals with different profiles and the synchronization of the applied field and current through the sample, as well as to acquire all signals, to trace the domain wall velocity and to compute the hysteresis loop for field or current dependence, using digital integration of the acquired signals. For the presented results, the hysteresis loop and DWV where recorded simultaneously. The pick-up coil signal can be amplified (up to 50000 times using an SR-560 Low-Noise Preamplifier) to measure domain wall displacement and magnetic properties in thin magnetic wires^14,32,39,40^.

For the presented results, using AC current excitation, we employed an arbitrary waveform build to generate three periods of a sinusoidal signal with equivalent frequency of 300 Hz (around 0.01s), followed by a pause of 1 second in which the signal is zero to allow heat dissipation. The signal was digitally generated, amplified and applied on the wire sample ends.
